# Targeting MD2 in prostate cancer bone metastasis: Mechanistic insights and therapeutic potential

**DOI:** 10.18632/oncoscience.647

**Published:** 2026-03-11

**Authors:** Melina A. Dattilo, Marina G. Ferrari, Alexis P. Jimenez-Uribe, Teresa Morales, Kyle T. Amber, Adrian P. Mansini

**Affiliations:** ^1^Universidad de Buenos Aires, Facultad de Medicina, Departamento de Bioquímica Humana, Buenos Aires, Argentina; ^2^CONICET – Universidad de Buenos Aires, Instituto de Investigaciones Biomédicas (INBIOMED), Buenos Aires, Argentina; ^3^Department of Urology, Rush University Medical Center, Chicago, IL 60612, USA; ^4^Department of Internal Medicine, Rush University Medical Center, Chicago, IL 60612, USA; ^5^Department of Dermatology, Rush University Medical Center, Chicago, IL 60612, USA; ^*^These authors contributed equally to this work

**Keywords:** prostate cancer, metastasis, MD2, biomarkers

## Abstract

Metastatic prostate cancer (PCa), especially when it involves the bone, remains a significant clinical challenge with limited therapeutic options. Our recent research identified Myeloid Differentiation Protein-2 (MD2/LY96) as a potential biomarker associated with poor prognosis and higher metastatic potential in PCa. In this Research Perspective, we build on those findings and present new preclinical data showing that pharmacological inhibition of MD2 markedly reduces tumor growth in a PCa mouse model of bone metastasis. Analysis of patient tumor tissues demonstrated that high MD2 expression is associated not only with metastasis but also with increased infiltration of T regulatory cells (Tregs) and myeloid-derived suppressor cells (MDSCs), indicating a role in promoting an immunosuppressive environment. Additionally, we show that soluble MD2 (sMD2) may serve as a non-invasive biomarker of metastatic burden and help predict resistance to poly ADP-ribose polymerase (PARP) inhibitor therapy.

This Research Perspective aims to consolidate mechanistic and preclinical evidence supporting MD2 as a driver of prostate cancer metastasis and to evaluate the therapeutic potential of pharmacological MD2 inhibition in a bone metastasis model.

These findings support MD2 as a novel therapeutic target and identify soluble MD2 as a promising predictive and prognostic biomarker in metastatic PCa, with mechanistic links to immune evasion and inflammatory signaling.

## MD2 IN PROSTATE CANCER: MORE THAN A BIOMARKER

MD2 is a co-receptor for Toll-like receptor 4 (TLR4), and it is essential for innate immune sensing [1]. However, beyond its canonical immunological role, MD2 is increasingly recognized as a modulator of tumor progression in various types of cancer [2–4]. In our recent publication [5], we demonstrated that high MD2 expression in prostate tumors correlates with increased metastatic potential and worse clinical outcomes, especially in patients with established metastases.

Additionally, transcriptomic profiling of highly expressing MD2 tumors revealed enrichment of inflammatory and epithelial-to-mesenchymal transition (EMT)-associated pathways, suggesting a functional role for MD2 in driving metastasis. High MD2 expression was also significantly associated with seminal vesicle invasion (SVI), extraprostatic extension (EPE), and the luminal molecular subtype, as we showed by Decipher genomic testing [5].

Mechanistically, we demonstrated that MD2 activates MAPK and NF-κB signaling, stabilizes HIF-1α and PKM2, and induces pro-metastatic factors including VEGF, MMP9, IL-8, and TGF-β1. Furthermore, we demonstrated that overexpression of MD2 promotes EMT and enhances the invasion, migration, and extravasation of prostate cancer cells. Importantly, we showed that MD2 is also released as a soluble protein (sMD2), detectable in the serum of patients with prostate cancer [5]. In contrast to PSA, sMD2 levels strongly correlate with metastatic burden, positioning it as a promising noninvasive biomarker for metastatic disease.

Here, we aimed to expand on our previous findings by evaluating the effects of selective MD2 inhibition in a preclinical model of prostate cancer bone metastasis, while incorporating mechanistic and immunological insights that support its therapeutic importance.

### New *In Vivo* evidence: MD2 inhibition suppresses bone metastatic growth

Since 90% of patients with metastatic PCa develop bone metastasis, we assessed the therapeutic potential of targeting MD2 *in vivo* using a preclinical model of PCa bone metastasis. Male nude mice were injected intratibially with luciferase-expressing PC3 cells. PC3 is a cell line derived from a bone metastasis of a grade IV prostatic adenocarcinoma. Once tumors were established, as confirmed by IVIS bioluminescence imaging, mice were randomized to receive either vehicle or the selective small molecule MD2 inhibitor, MD2-int-1 (MedChemExpress), for three weeks.

### Key findings (Figure 1)

Tumor progression, monitored by IVIS Spectrum imaging, was significantly reduced in mice treated with the MD2 inhibitor compared to vehicle controls. The bioluminescence signal, which indicates tumor burden, remained stable or decreased over time in the treated group, while it increased sharply in the control group. Quantitatively, the mean average radiance (photons/sec/cm2/sr) at week 3 (endpoint) was decreased by approximately 75% in MD2 inhibitor–treated mice compared to controls (1.2 × 10^6^ ± 0.5 × 10^6^ vs. 4.7 × 10^6^ ± 0.4 × 10^6^; *p* < 0.001, two-way ANOVA with repeated measures). The treatment was well tolerated, with no signs of toxicity or significant weight loss (<5%) observed during the study.

**Figure 1:**
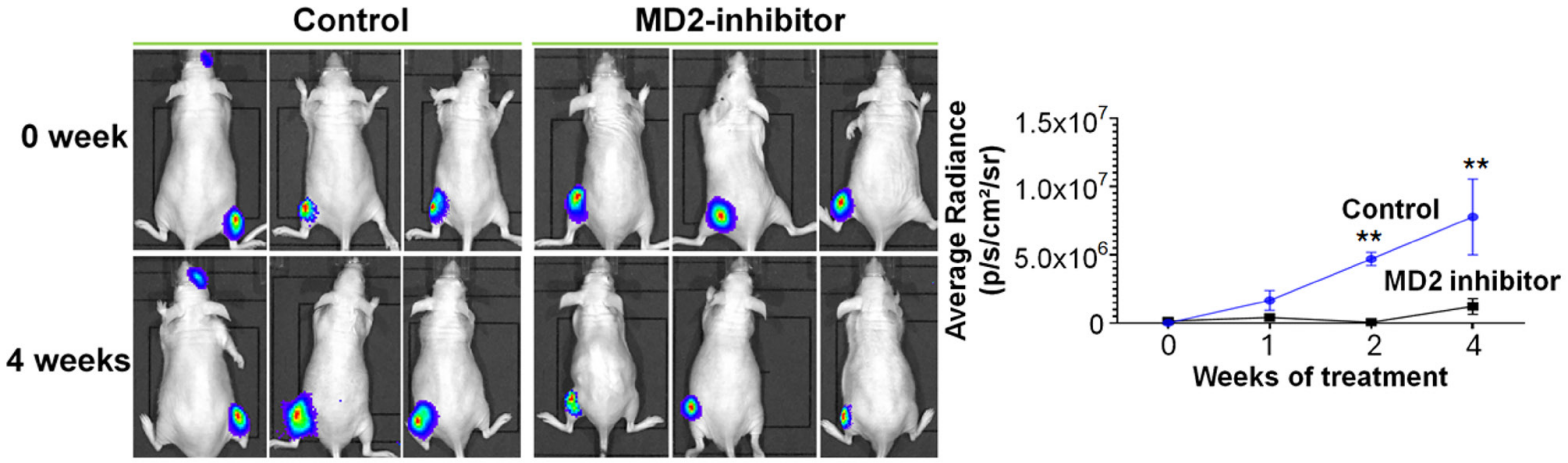
Effectiveness of MD2 inhibitor therapy on tumor growth in a prostate cancer bone metastasis model. (**A**) Representative bioluminescence images of vehicle-treated and MD2 inhibitor– treated mice before and after treatment, obtained using the IVIS Spectrum imaging system. (**B**) Quantification of average radiance (photons/sec/cm2/sr) over the 3-week treatment period. Data are presented as mean ± SEM; *p* < 0.01, two-way ANOVA with repeated measures.

## MECHANISTIC INSIGHTS AND CLINICAL RELEVANCE

The mechanisms by which MD2 promotes bone metastasis may include:

1. Activation of the TLR4-MD2 signaling axis promoting EMT and invasion.
2. Detection of danger-associated molecular patterns (DAMPs) such as oxidized lipoproteins or alarmins that promote EMT and invasion [6, 7].
3. Crosstalk with osteoclasts, promoting a bone immunosuppressive environment, bone resorption, and tumor colonization.
4. The induction of HMGB1 release, as demonstrated in our *in vitro* experiments, facilitates a pro-metastatic tumor microenvironment.

**Figure 2:**
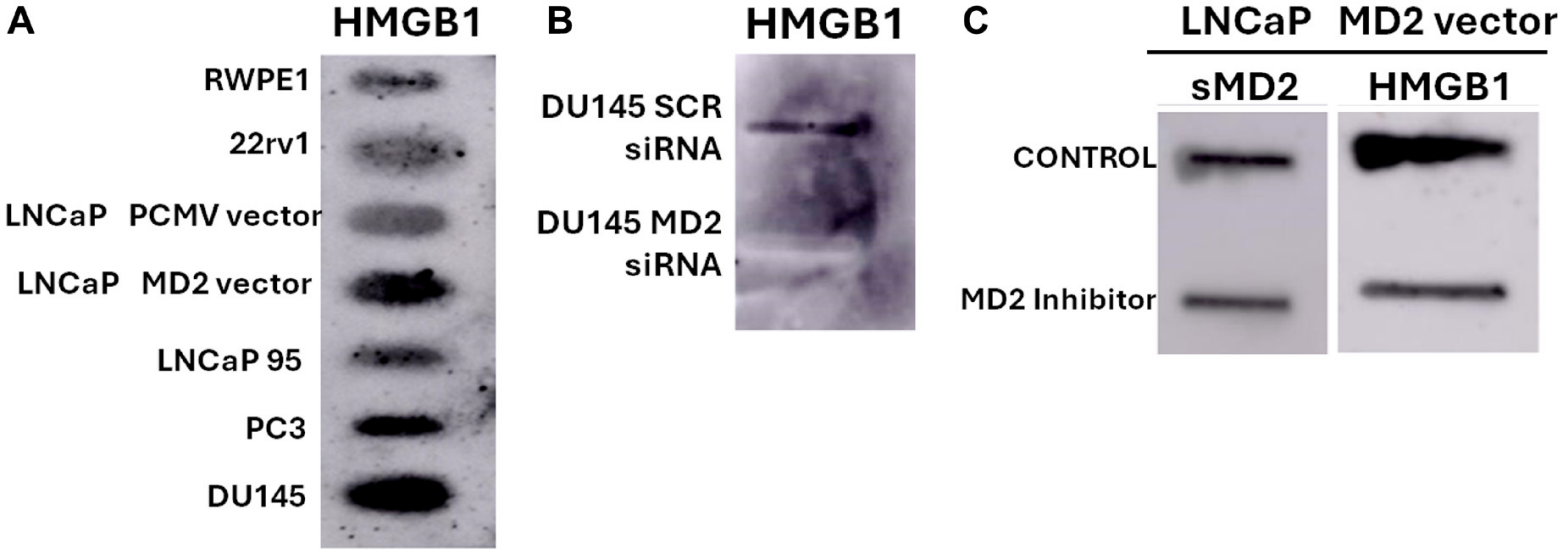
Dot blot analysis of HMGB1 and soluble MD2 (sMD2) in conditioned media (CM) from prostate cancer cell lines. (**A**) Detection of HMGB1 in CM from a panel of prostate cancer (CaP) cell lines, including LNCaP control (PCMV vector) and MD2-overexpressing cells (M2 vector). (**B**) HMGB1 levels in CM from DU145 control cells (SCR-siRNA) and MD2-silenced cells (MD2 siRNA). (**C**) Levels of soluble MD2 (sMD2) and HMGB1 in CM from LNCaP cells overexpressing MD2 after treatment with the MD2 inhibitor (MD2-int-1) or vehicle control for 24 h.

HMGB1 is a well-characterized alarmin released during cellular stress or necrosis and is known to promote immune cell recruitment, EMT, and resistance to apoptosis [8, 9]. Notably, dot blots using conditioned media from PCa cells showed that metastatic cells expressing MD2 (PC3, DU145) release HMGB1, and ectopic expression of MD2 in LNCaP cells leads to HMGB1 secretion (Figure 2A). This suggests a feed-forward inflammatory loop mediated by MD2 that promotes metastatic capacity. Additionally, we found that knocking down MD2 in DU145 cells decreased HMGB1 release, and pharmacological inhibition of MD2 in LNCaP cells reduced the secretion of sMD2 and HMGB1 (Figure 2B, 2C). Therefore, targeting MD2 could be a new approach to suppress both inflammation and metastasis in prostate cancer.

**Figure 3:**
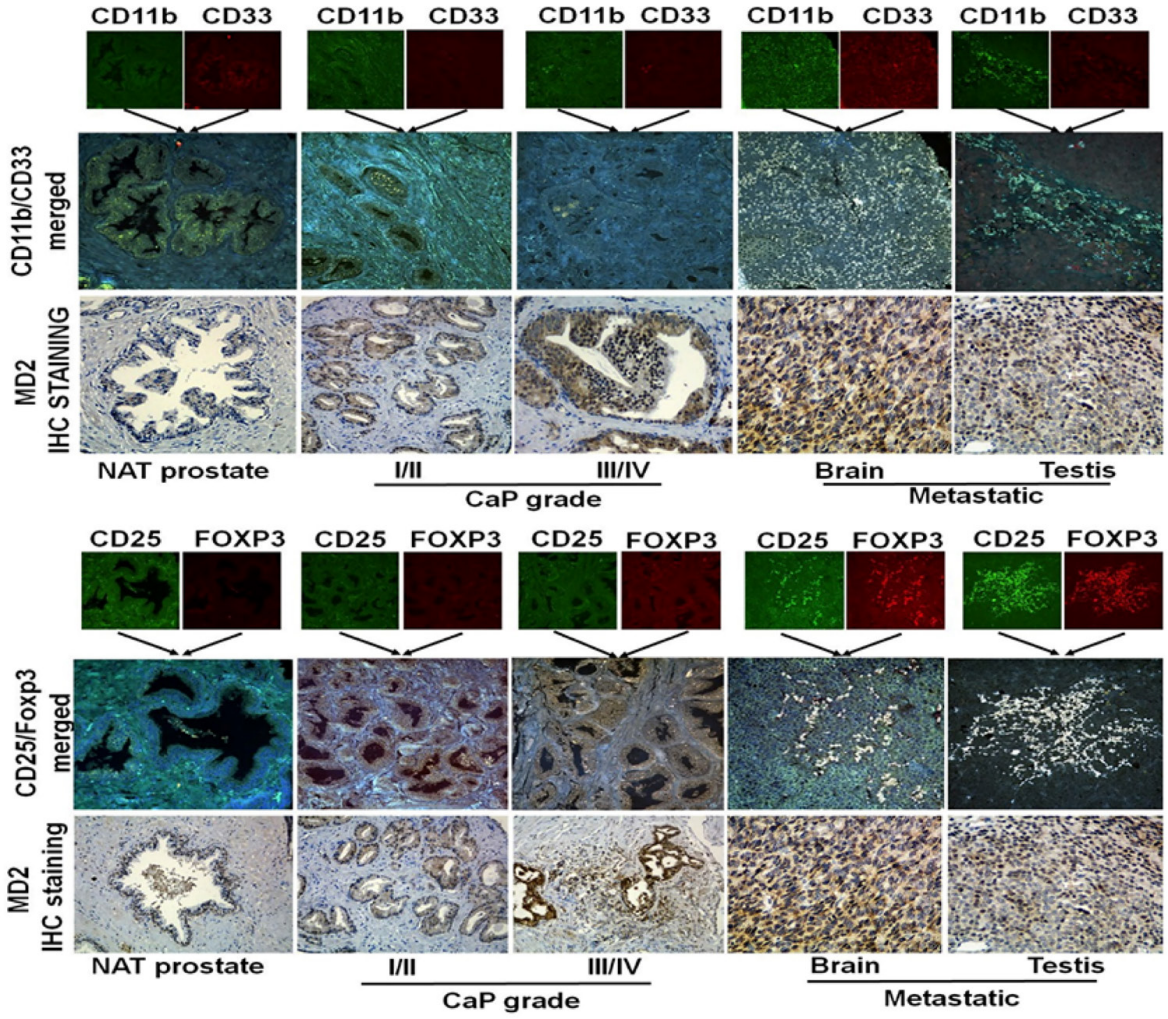
Immunohistochemistry (IHC) and Immunofluorescence (IF) analysis of MD2 expression and immune cell infiltration in prostate cancer tissues. (Upper panel) Representative IF images showing myeloid-derived suppressor cells (MDSCs; CD11b/CD33) and corresponding IHC staining for MD2 in patient tissues with varying prostate cancer (PCa) grades, metastatic lesions, and histologically normal adjacent tissue (NAT). (Lower panel) Representative IF images showing regulatory Tcell (Treg; CD25/Foxp3) infiltration and corresponding IHC staining for MD2 in PCa tissues with different tumor grades, metastasis, and NAT.

Moreover, our immunohistochemical and immunofluorescence analysis of human PCa tissues revealed that high MD2 expression is associated with increased infiltration of immunosuppressive cell populations, including T regulatory cells (Tregs) and myeloid-derived suppressor cells (MDSCs) (Figure 3). This observation suggests that MD2 may contribute to shaping an immunosuppressive tumor microenvironment, further promoting tumor immune evasion and metastatic progression.

**Figure 4:**
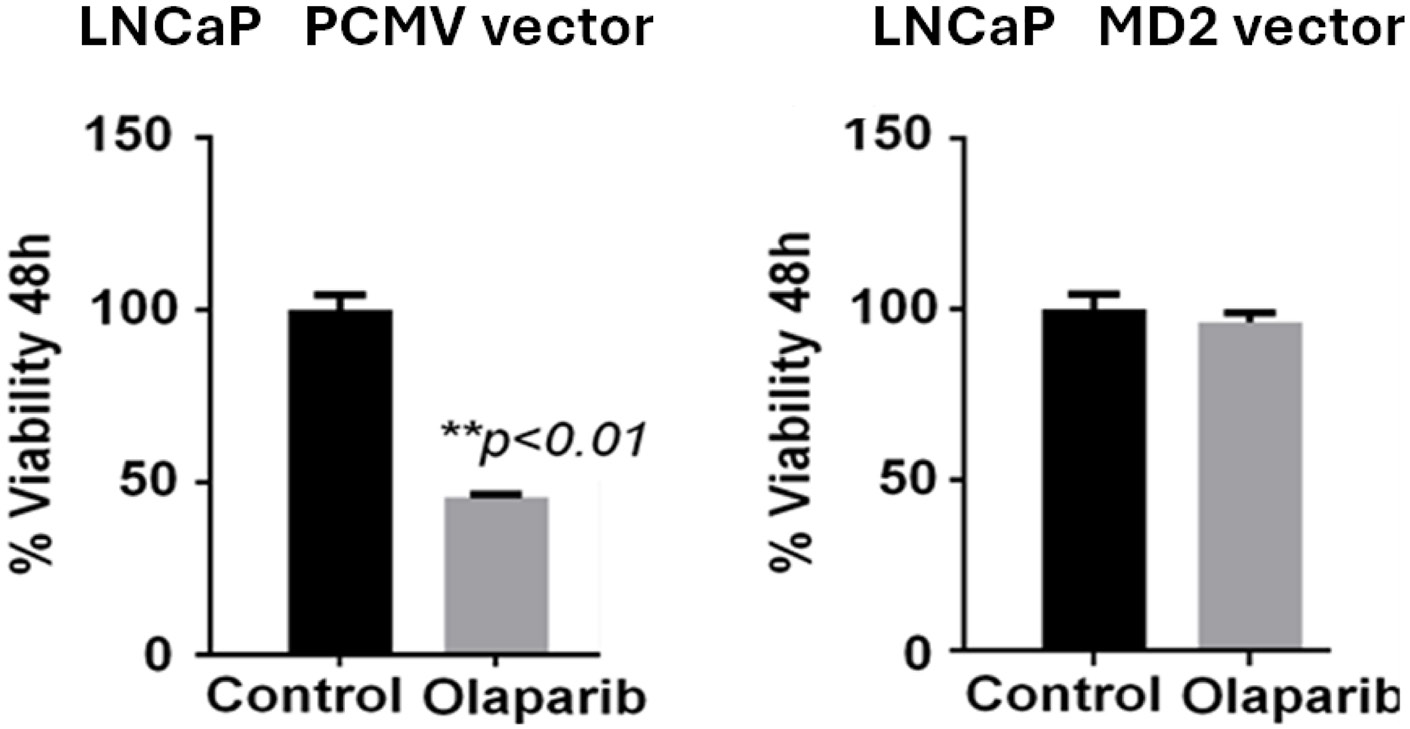
Effect of Olaparib treatment on cell viability in MD2-overexpressing prostate cancer cells. Bar graphs show the PARP-1 inhibitor Olaparib (10 μM, 48 h) on LNCaP control cells (PCMV vector) and MD2-overexpressing cells (MD2 vector). Data are presented as mean ± SEM; *p* < 0.01, Student’s t-test.

Additionally, preliminary data from LNCaP cells engineered to overexpress MD2 (LNCaP MD2 vector) show decreased sensitivity to Olaparib, a PARP-1 inhibitor (Figure 4), suggesting a potential role for MD2 in the DNA damage response and therapeutic resistance. Cell viability assays revealed that after 48 hours of Olaparib exposure (10 μM), viability decreased by 44 ± 5% in control LNCaP cells but only 18 ± 4% in MD2overexpressing cells (*p* < 0.01, Student’s *t*-test), confirming a partial resistance phenotype.

These results suggest that MD2 overexpression may activate alternative survival pathways or reduce DNA repair stress signals, leading to resistance even when PARP1 is inhibited. If confirmed, MD2 could serve as a predictive biomarker for response to PARP-1 inhibitors and a target for combined therapy.

These findings collectively emphasize the diverse oncogenic functions of MD2, recognizing it as a central link that connects inflammation, immune evasion, tumor progression, and therapy resistance in PCa. Targeting MD2 could allow for multifaceted inhibition of these processes. Unlike broad TLR4 inhibition, targeting MD2 specifically may produce more targeted anti-tumor effects with fewer systemic immune-related side effects. Although MD2 is also found in innate immune cells, strategies that selectively target it on tumor cells may reduce systemic immunosuppression. Additionally, the presence of circulating sMD2 could serve as a companion biomarker to help guide treatment choices and track therapeutic response. These findings provide a foundation for clinical translation, including biomarker-based patient stratification and the development of new therapies.

## ADDITIONAL MECHANISTIC CONSIDERATIONS

MD2 may promote an immunosuppressive tumor microenvironment by activating the TLR4–MyD88–NF-κB axis, leading to the upregulation of pro-inflammatory cytokines (IL-6, IL-8, TNF-α) and chemokines (CCL2, CCL5, CXCL8) that recruit regulatory T cells (Tregs) and myeloid-derived suppressor cells (MDSCs) [10, 11]. This paracrine signaling cascade can reinforce a chronic inflammatory milieu while simultaneously suppressing cytotoxic T cells responses, thereby favoring tumor progression and metastatic dissemination. Additionally, MD2-dependent TLR4 signaling may intersect with metabolic and hypoxia-responsive pathways, such as HIF-1α activation and PKM2 stabilization, which further enhance glycolytic flux, angiogenic signaling, and immune evasion [5, 12]. Future studies employing metabolic profiling and single-cell transcriptomic approaches will be essential to dissect how MD2 integrates inflammatory and metabolic cues to sustain tumor-associated immunosuppression.

## BROADER IMPLICATIONS IN PROSTATE CANCER THERAPY

The present findings position MD2 within a broader therapeutic framework for metastatic prostate cancer. By linking inflammatory and immune-escape pathways, MD2 could complement existing therapeutic strategies such as androgen deprivation therapy (ADT), PARP inhibition, and immune checkpoint blockade. Targeting MD2 might enhance treatment efficacy in aggressive or therapy-resistant disease, while circulating sMD2 could serve as a companion biomarker to stratify patients and monitor treatment response.

## LIMITATIONS

Although these preclinical findings support the biological and therapeutic importance of MD2 in prostate cancer bone metastasis, several limitations should be recognized. The sample size in the animal study was small, limited to a single prostate cancer cell line (PC3) and a single MD2 inhibitor compound. Additional validation across various approaches, including genetic knockdown models, different cell lines, and Patient-derived xenografts, will be necessary to confirm the broader applicability of these findings. Moreover, while our immunohistochemical analyses suggest a link between MD2 expression and immunosuppressive cell infiltration, the exact mechanisms underlying this association remain to be clarified. Lastly, the potential of soluble MD2 as a biomarker needs to be tested in clinical cohorts to assess its predictive and prognostic value.

## FUTURE DIRECTIONS

Building on our current findings, several futures research avenues are warranted:

### Combination therapies with MD2 inhibitors

- Explore the synergistic potential of MD2 inhibition combined with androgen deprivation therapy (ADT), especially in castration-resistant prostate cancer (CRPC).
- Assess the effectiveness of MD2 inhibitors combined with immune checkpoint blockade (e.g., anti-PD-1/PD-L1), especially in metastatic tumors with immunosuppressive microenvironments.
- Assess whether pharmacological inhibition of MD2 sensitizes resistant PCa tumors to the PARP-1 inhibitor Olaparib.

### Overcoming therapeutic resistance

- Expand on preliminary findings showing that MD2 overexpression in LNCaP cells confers resistance to PARP-1 inhibitors, possibly via modulation of DNA repair or antiapoptotic pathways. These results suggest a role for MD2 in shaping drug sensitivity and resistance, independent of BRCA status.
- Evaluate whether MD2 expression is associated with clinical resistance to PARP inhibitors in patient-derived samples.

### HMGB1 as a co-target

- Explore the MD2-HMGB1 axis as a new therapeutic target. Inhibiting MD2 could reduce pro-inflammatory signaling and prevent metastatic progression.
- Assess whether HMGB1 release is necessary for MD2-mediated EMT, bone colonization, or immunomodulation.

### MD2 as a complementary diagnostic and prognostic marker

- Validate sMD2 as a predictive and prognostic serum biomarker in prospective patient cohorts.
- Correlate sMD2 levels with tumor burden, treatment response, and survival across different therapy types (ADT, chemotherapy, PARP inhibitors, immunotherapy).

### Development of advanced therapeutic modalities

- Design antibody-based inhibitors, nanobody constructs, or PROTACs for selective degradation of MD2.
- Explore targeted drug delivery systems using lipid nanoparticles or exosomes to specifically deliver MD2-targeting agents to bone metastatic lesions.
- Evaluate the safety and immunogenicity of the chosen therapy for long-term use.

### Broader implications of MD2 signaling

- Examine the role of MD2 in other bone-tropic cancers such as breast, renal, and multiple myeloma.
- Determine whether MD2 contributes to bone tropism through osteoclast activation, matrix remodeling, or niche colonization.

### Single-cell and spatial profiling

- Use spatial transcriptomics and single-cell RNA-seq to characterize MD2- expressing cells within the tumor microenvironment and bone metastatic niche.
- Identify cell-type–specific contributions to the MD2-HMGB1-EMT axis and immune evasion mechanisms.
- Further characterize the association between MD2 expression and immunosuppressive cell infiltration (e.g., Tregs, MDSCs) using multiplex IHC, spatial transcriptomics, and scRNA-seq.

## MATERIALS AND METHODS

### Cell line and animal model

Human prostate cancer PC3 cells stably expressing luciferase and LNCaP cells were cultured as previously described [5]. Transfections, dot blots, immunoblots, and viability were performed as described [5, 13]. For bone metastasis modeling, 6-week-old male athymic nude mice (Foxn1nu, Jackson Lab) were used.

### Ethical statement: Animal studies

All animal experiments complied with institutional and national guidelines. Animal studies received approval from the Institutional Animal Care and Use Committee (IACUC) at Rush University (Protocol ID: 23-038).

### Intra-tibial injection and tumor establishment

Mice were anesthetized and injected intratibially with 1 × 10^5^ PC3-Luc cells in 20 μL PBS into the right hind limb. Tumor growth was monitored weekly using the IVIS Spectrum imaging system after intraperitoneal injection of D-luciferin (150 mg/kg).

### Treatment regimen

Upon confirming the localized bioluminescence signal (day 7–10 post-injection), mice were randomized into two groups: vehicle control (*n* = 5), receiving 10% DMSO/90% corn oil; and MD2 inhibitor group (*n* = 5), receiving MD2-int-1 via intraperitoneal injection at 10 mg/kg, three times a week for three weeks. Body weight and clinical condition were monitored throughout the study.

### Dot blot, immunofluorescence, immunohistochemistry, and viability assays

They were performed as previously described [5, 14].

### Statistical analysis

Quantitative data from bioluminescence imaging (total flux [photons/sec]) were analyzed using GraphPad Prism v10. Longitudinal differences in tumor growth were evaluated with a two-way ANOVA with repeated measures, followed by Bonferroni post-hoc tests. Final tumor burdens at the endpoint were compared using an unpaired two-tailed Student’s *t*-test. A *p*-value < 0.05 was considered statistically significant.

## CONCLUSIONS

This perspective highlights MD2 as a promising biomarker and therapeutic target in prostate cancer metastasis, particularly within the bone microenvironment. Our new *in vivo* data reinforce the rationale for therapeutic inhibition of MD2 and underscore the need for continued preclinical development and future clinical investigation.

Collectively, these findings highlight MD2 as a key regulator connecting inflammation, immune evasion, and metastatic progression in prostate cancer. Targeting MD2 could provide a new mechanism-based treatment approach, potentially improving outcomes for patients with bone-predominant or therapy-resistant disease, while circulating soluble MD2 might serve as a minimally invasive biomarker to help guide treatment decisions and monitor responses.
